# Epidemiological association and machine learning-based prediction of lung cancer risk linked to long-term lagged satellite-derived PM_2.5_ in China

**DOI:** 10.3389/fpubh.2025.1536509

**Published:** 2025-05-30

**Authors:** Feiran Wei, Shijun Yang, Huiying Wang, Meng Zhao, Jinyi Zhou, Xiaobing Shen, Renqiang Han, Gaoqiang Fei

**Affiliations:** ^1^Key Laboratory of Environmental Medicine Engineering, Ministry of Education, School of Public Health, Southeast University, Nanjing, China; ^2^Guangxi Meteorological Observatory, Nanjing, China; ^3^Lianyungang Meteorological Bureau, Lianyungang, China; ^4^Department of Epidemiology and Biostatistics, School of Public Health, Southeast University, Nanjing, China; ^5^Jiangsu Provincial Center for Disease Control and Prevention (Jiangsu Provincial Academy of Preventive Medicine), Nanjing, China; ^6^Department of Public Health, Jiangsu Cancer Hospital, The Affiliated Cancer Hospital of Nanjing Medical University, Jiangsu Institute of Cancer Research, Nanjing, China

**Keywords:** PM_2.5_, lung cancer, long-term exposure, machine learning, prediction model, public health

## Abstract

**Objectives:**

This study investigated association between long-term PM_2.5_ exposure and lung cancer incidence, focusing on Jiangsu Province, China. We aimed to explore the effects of historical PM_2.5_ with time lags and build a prediction model using machine learning methods.

**Study design:**

An ecological epidemiology study.

**Methods:**

Lung cancer incidence data from Jiangsu Province (2014–2018) were combined with annual PM_2.5_ concentration data from satellite sources for the previous 10 years (lag 0 to lag 9). Correlation and grey correlation analyses were performed to evaluate the lagged relationship between PM_2.5_ exposure and lung cancer incidence. To address the multicollinearity problem in the data, ridge regression, support vector regression, and back propagation artificial neural network were employed. The combined prediction model was constructed using the optimal weighting method.

**Results:**

The incidence of lung cancer was significantly correlated with PM_2.5_ concentration at different historical time points, with the strongest correlation at lag 9. The combined prediction model that integrates multiple prediction methods showed higher accuracy and reliability in predicting lung cancer incidence than a single model.

**Conclusion:**

Long-term exposure to PM_2.5,_ especially exposure with a long lag time, is closely related to lung cancer incidence. The integrated machine learning prediction model can be used as a reliable tool to assess the health risks of air pollution.

## 1 Introduction

According to the latest cancer statistics released by the International Agency for Research on Cancer, lung cancer remains one of the most common malignant tumors, accounting for 11.4% of new cancer cases and 18.0% of cancer-related deaths worldwide in 2020 (1, 2). Among males, lung cancer ranks as the leading cause of cancer incidence and mortality. In females, lung cancer ranks third in incidence after breast and colorectal cancers, and second in mortality, only preceded by breast cancer (1). In China, lung cancer tops the list of cancer types in terms of both incidence and mortality, with over 700,000 deaths attributed to lung cancer in 2020, imposing a significant disease burden (3).

Air pollution, a major threat to public health, is closely associated with an increased risk of lung cancer (4). As a major component of air pollution, fine particulate matter (PM_2.5_) carries various harmful substances and can be directly inhaled and deposited throughout the respiratory tract, including the deepest alveolar epithelial cells, thereby inducing lung injury or respiratory dysfunction and further increasing the risk of lung cancer (5). In our previous study, we delved into the epidemiological trends of PM_2.5_-related lung cancer in China using global burden of disease data (6). It was found that while disability-adjusted life years (DALYs) attributed to lung cancer caused by household pollution sources showed a downward trend, those caused by air pollution sources increased significantly, highlighting the significant role of outdoor particulate pollution in increasing the burden of lung cancer. Therefore, this study focuses on investigating the potential association between PM_2.5_ concentrations in the outdoor environment and lung cancer incidence.

The impact of annual average PM_2.5_ concentrations in the atmosphere on lung cancer incidence may be a long-term accumulative process, implying that the incidence of lung cancer may be related to long-term exposure to PM_2.5_ over years rather than directly linked to the current PM_2.5_ concentration (7–9). Although no study has yet definitively determined the exact duration of the sustained impact of PM_2.5_ concentrations on lung cancer incidence, several studies have suggested that there exists a time lag effect between lung cancer incidence and exposure to air pollution concentrations, with a latency period of at least 7–8 years for lung cancer caused by atmospheric PM_2.5_ (9–11). Based on this research background, this study analyzes lung cancer incidence data from selected regions in Jiangsu Province from 2014 to 2018, combined with satellite-derived annual average PM_2.5_ concentration data from the past 10 years (including the current year), to reveal the potential association and lag effect between PM_2.5_ concentrations and lung cancer incidence, and to construct corresponding machine learning prediction models.

## 2 Methods

### 2.1 Data sources

The data sources for this study comprise two main parts: lung cancer incidence rates and PM_2.5_ concentrations.

The lung cancer incidence data were obtained from the official cancer registry of the Jiangsu Provincial Center for Disease Control and Prevention (CDC), covering the period from 2014 to 2018 in multiple regions of Lianyungang and Suzhou cities. This cancer registry operates under standardized national protocols for data collection, verification, and quality control, ensuring high levels of reliability and completeness. All cases were classified according to the International Classification of Diseases for Oncology, 3rd Edition (ICD-O-3) (12) 和 ICD-10 (13) coding standards, encompassing lung cancer cases within the range of C34.0-C34.9.

The PM_2.5_ concentration data were sourced from a satellite-derived dataset developed by the School of Medicine at Washington University in St. Louis (14, 15). This dataset combines information from multiple sources, including satellite remote sensing, chemical transport models, and ground-based monitoring stations, to estimate ground-level PM_2.5_ concentrations with high spatial and temporal resolution. The integration of diverse data sources, along with the application of advanced statistical modeling techniques, ensures the accuracy and robustness of PM_2.5_ estimates. The dataset has been widely validated and applied in international environmental health studies, supporting its credibility and applicability in this research (16–18).

In this study, we utilized Python and ArcGIS10.5 software to extract high-resolution annual average PM_2.5_ concentration data for each study region during the corresponding years of lung cancer incidence (2014–2018), as well as for the previous nine years (2005–2018). These exposure values were labeled as lag0 to lag9, representing cumulative exposure windows and serving as key indicators for evaluating the long-term impact of PM_2.5_ on lung cancer incidence.

### 2.2 Statistical analysis and modeling

#### 2.2.1 Correlation and grey relational analysis

In this study, we first employed correlation analysis to evaluate the relationship between lung cancer incidence and PM_2.5_ concentrations from different lag years (lag0 to lag9). To determine the appropriate correlation method, we conducted a Shapiro–Wilk test to assess the normality of both variables across each lag year. When both variables exhibited a normal distribution (*p* > 0.05), we applied the Pearson correlation coefficient; otherwise, the Spearman rank correlation was used.

Additionally, we employed grey relational analysis to assess the degree of similarity in trends between lung cancer incidence and PM_2.5_ concentrations across different lag years. Grey relational analysis helps identify which lag periods show the strongest relational closeness with the observed incidence patterns, thereby revealing the most influential exposure windows for lung cancer risk.

#### 2.2.2 Collinearity test and ridge regression model

Before conducting multivariate statistical analysis, we calculated the correlation coefficient matrix, variance inflation factor (VIF), tolerance, eigenvalues, and condition index to examine the correlation and collinearity among PM_2.5_ concentrations from different lag years, ensuring the stability of model construction. To address potential multicollinearity issues, we adopted the ridge regression model to assess the relationship between PM_2.5_ concentration lag factors and lung cancer incidence. The optimal regularization parameter was selected based on the ridge trace plot to guarantee the predictive performance of the model.

#### 2.2.3 Support vector regression (SVR) model

Using the SVR model, we constructed lung cancer incidence prediction models with four different kernel functions (linear, Sigmoid, RBF, and polynomial). By comparing the mean squared error (MSE) and R^2^ score, the optimal kernel function was selected, and feature importance analysis was performed based on this model.

#### 2.2.4 The back propagation artificial neural network (BP-ANN)

The BP-ANN was employed to further explore the relationship between PM_2.5_ concentration lag factors (lag0 to lag9) and lung cancer incidence. The BP-ANN learns complex patterns in the data for prediction by simulating the working mechanism of human brain neurons. We set three hidden layers, optimizing the number of nodes in each hidden layer between 5 and 20 to balance the complexity and generalization ability of the model. The input layer contains 10 nodes, corresponding to the 10 PM_2.5_ concentration lag factors. The ReLU activation function was chosen to improve the learning efficiency and prediction accuracy of the network.

#### 2.2.5 Combined prediction model

To further enhance prediction accuracy and stability, we constructed a combined prediction model that integrates the ridge regression model, SVR, and BP-ANN. The weights of each individual model were determined using the standard deviation method, reciprocal variance method, and optimal weighting method. The prediction results of different models were then fused to reduce errors and uncertainties. We comprehensively evaluated the performance of the combined prediction model using indicators such as the mean absolute error (MAE), MSE, mean absolute percentage error (MAPE), and Theil’s U statistic.

## 3 Results

### 3.1 Correlation analysis between lung cancer incidence and PM_2.5_ concentration

As shown in Supplementary Table S1, both the lung cancer incidence rates and PM_2.5_ concentration data across lag0 to lag9 passed the Shapiro–Wilk normality test (*p* > 0.05). Therefore, the Pearson correlation coefficient was used to assess the correlation between lung cancer incidence and PM_2.5_ concentrations at each lag year.

The results of the univariate correlation analysis (Supplementary Table S2) showed that lung cancer incidence was significantly correlated with PM_2.5_ concentrations at lag3, lag4, lag5, lag7, lag8, and lag9, with the strongest correlation observed at lag9. In contrast, some lag years, such as lag6, did not show statistically significant associations (*p* > 0.05). This suggested that the association between PM₂.₅ exposure at a single lag year and lung cancer incidence may not always be strong. It is important to note that this univariate analysis serves as a preliminary exploration and may not fully capture the complex and cumulative nature of air pollution’s impact on cancer development.

The grey relational analysis (Table 1) provided complementary insights, revealing consistently strong relational degrees between PM_2.5_ concentrations and lung cancer incidence across all lag years, with lag3, lag8, and lag9 showing the highest overall association. This suggests that the effect of PM_2.5_ exposure may span multiple years and reflect long-term cumulative risk rather than isolated time points.

**Table 1:** Grey relational analysis results of lung cancer incidence.

	2014	2015	2016	2017	2018	Overall
lag9	0.678490	0.558407	0.612215	0.583663	0.514258	0.693152
lag8	0.640411	0.551083	0.614859	0.577470	0.536022	0.694986
lag7	0.675807	0.555470	0.618089	0.589302	0.515062	0.684973
lag6	0.668597	0.549478	0.627684	0.582683	0.575642	0.685135
lag5	0.669268	0.547409	0.618063	0.631675	0.534171	0.679667
lag4	0.656381	0.544861	0.657607	0.587821	0.538573	0.690066
lag3	0.648477	0.565997	0.620572	0.582679	0.520465	0.699169
lag2	0.630912	0.543396	0.612611	0.591382	0.598212	0.684857
lag1	0.643389	0.534456	0.633955	0.632408	0.622666	0.648438
lag0	0.645247	0.561204	0.648804	0.663933	0.611975	0.640340

Overall, these analyses offer preliminary evidence of temporal associations between long-term PM_2.5_ exposure and lung cancer incidence, supporting further exploration using multivariate modeling approaches to capture the potential cumulative effects of air pollution across multiple time periods.

### 3.2 Prediction of lung cancer incidence using machine learning models

#### 3.2.1 Feature analysis of influencing factors for lung Cancer incidence

The correlation coefficient matrix of the 10 influencing factors (lag0 to lag9) is presented in Supplementary Table S3, revealing varying degrees of correlation among these factors. For example, the correlation coefficient between lag1 and lag0 was as high as 0.951, and the correlation coefficient between lag9 and lag4 reached 0.787, indicating a strong positive correlation. Therefore, before constructing the prediction model, it is necessary to perform a collinearity test to ensure the selection of an appropriate model and the robustness and validity of the prediction results.

Supplementary Tables S4, S5 present the results of the collinearity test. The results showed that the VIF values of most factors exceeded 10, indicating significant collinearity. Specifically, the VIF values of lag1 and lag0 reached 69.89 and 55.65, far exceeding the conventional threshold of 10 for identifying significant collinearity. Correspondingly, the tolerance values of these variables were extremely low, with the tolerance of lag1 and lag0 being only 0.014 and 0.018, respectively, further confirming the collinearity issue among the variables. In Supplementary Table S5, we observed that as the dimension increased, the eigenvalues gradually decreased. The eigenvalues from the third to the eleventh dimension were almost zero. Simultaneously, the condition indices of these dimensions exceeded 30 and increased with the increase in dimension. These observations indicate that there is significant multicollinearity among the influencing factors, which needs to be considered in subsequent analysis and model building.

Given the significant collinearity issue revealed in the aforementioned analysis among the influencing factors, to enhance the prediction accuracy of the lung cancer incidence prediction model, this study will employ Ridge Regression, SVR, and BP-ANN as subsequent modeling methods. These methods exhibit high robustness in handling datasets with collinear variables, effectively reducing the negative impact of collinearity on the performance of prediction models, thus optimizing the prediction effect of lung cancer incidence.

#### 3.2.2 Prediction of lung cancer incidence rate model based on ridge regression

**Figure 1:**
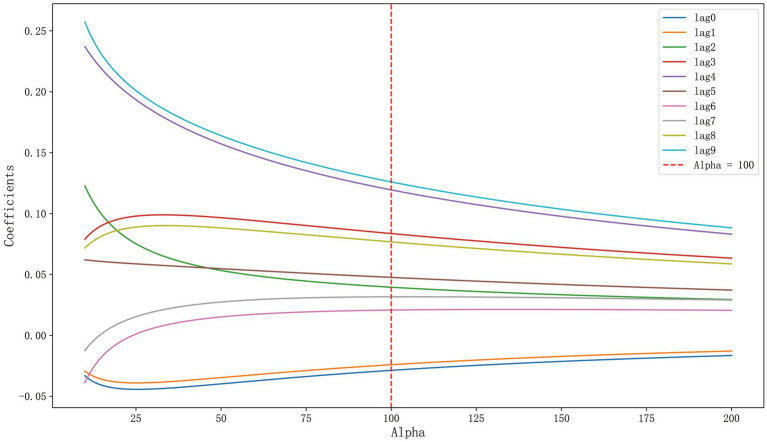
Ridge trace plot of lung cancer incidence.

A ridge trace plot was initially constructed to observe the impact of different ridge parameters k on the regression coefficients. Figure 1 demonstrated the changing trends of each coefficient under varying degrees of regularization, specifically how the coefficient of each variable (e.g., lag0 to lag9) varied with the change in k values. When the ridge parameter k exceeded 100, we observed a stable pattern in the regression coefficients of the 10 influencing factors, including lag0 to lag9. However, considering that an increase in k was accompanied by an increase in MSE, a higher ridge parameter k might weaken the goodness of fit of the regression equation. Therefore, we selected the coefficients of influencing factors under k = 100 as the parameters for the ridge regression model, aiming to balance the bias and variance of the model. At this point, the coefficients were stable and the MSE was within an acceptable range, ensuring that the goodness of fit of the model was not compromised by an excessively high ridge parameter.

As shown in Supplementary Table S6, when k = 100, the modified VIF values of all variables were below 5, indicating effective control of collinearity. It was evident that the selected ridge parameter k = 100 significantly reduced multicollinearity in the model. At this time, the MSE of the model was 0.8290, and R^2^ was 0.3193.

**Figure 2:**
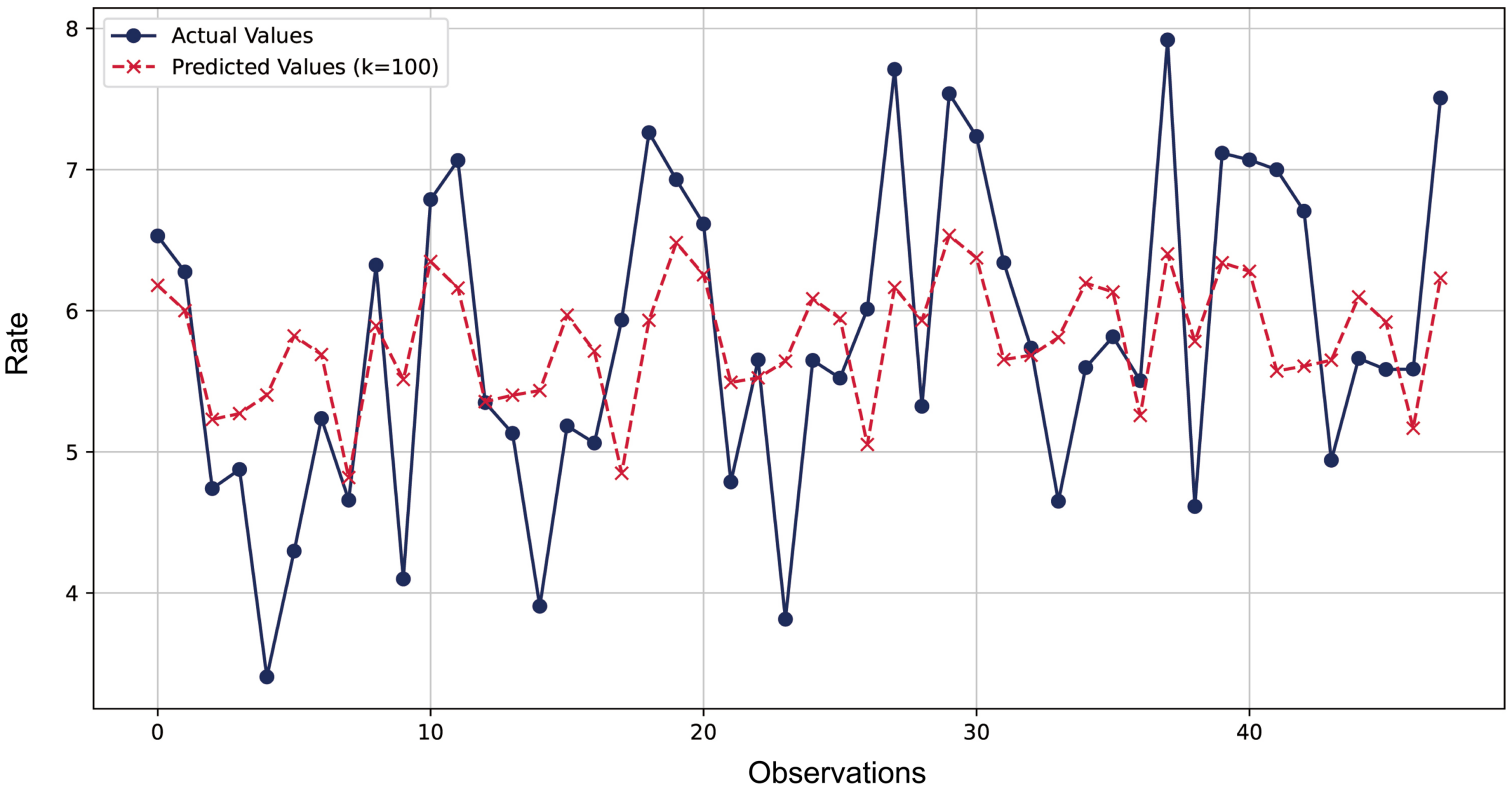
Comparison of ridge regression prediction and actual observed values for lung cancer incidence.

Figure 2 compared the predicted values of the ridge regression model with the actual values. The blue lines and dots represented the actual observations, while the red dashed lines and crosses represented the predicted values of the ridge regression model. It could be observed from the figure that the model’s predictions were very close to the actual observations at most data points, with the relative error basically controlled within 10%. The error ratios of most data points were concentrated in a lower range (below 3%), indicating that the model provided relatively accurate predictions for most data points and demonstrated good predictive performance.

#### 3.2.3 Prediction of lung cancer incidence rate model based on SVR

Supplementary Table S7 presents the performance evaluation results of various kernel function models. The MSE of the linear kernel model was 2.6861 with an R^2^ score of −0.5603, indicating that its predictive performance not only failed to surpass the baseline prediction using simple mean but was even worse. The performance of the polynomial kernel model was even more unsatisfactory, with an MSE of 3.6491 and an R^2^ score plummeting to −1.1196, suggesting its prediction effectiveness fell far below the baseline level. In contrast, the performance of the Sigmoid kernel model showed improvement, with the MSE decreasing to 1.8743 and the R^2^ score rising to −0.0887. While this still indicated a relatively weak predictive capability, it represented significant progress compared to the linear kernel model. Among all kernel functions, the radial basis function (RBF) kernel model exhibited the optimal performance, with its MSE reduced to 0.8860 and the R^2^ score increasing to 0.4854, indicating that the model was able to capture data variability effectively and make accurate predictions.

**Figure 3:**
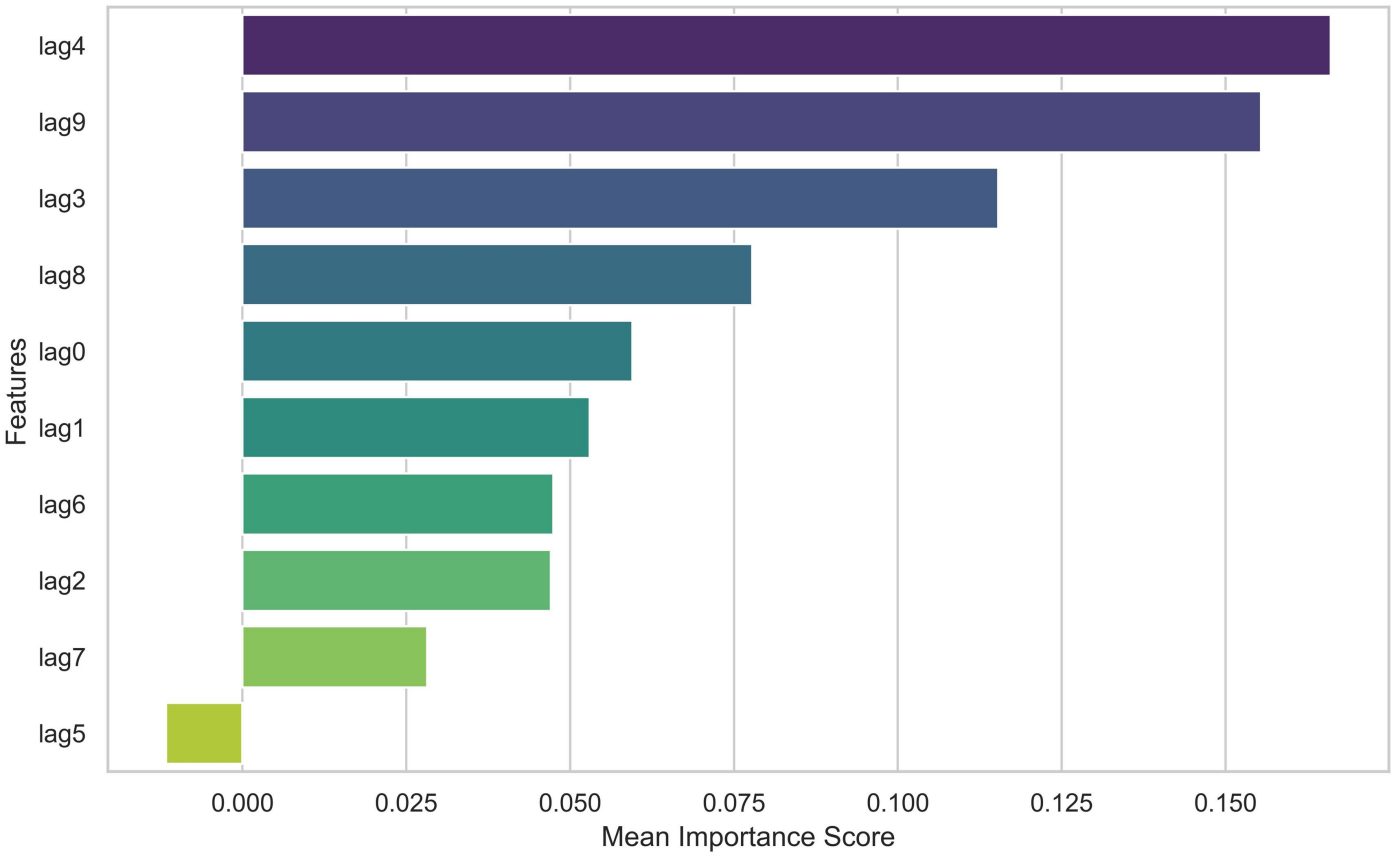
Feature importance scores of various factors in the SVM model for lung cancer incidence prediction.

Based on the model performance evaluation results, we selected the RBF kernel for training the SVR model and further calculated the importance of each factor in the model. As shown in Figure 3, it can be observed that the contribution of each lag variable (lag0 to lag9) to the model’s prediction of lung cancer incidence rate varies. The lag4 feature had the highest average importance score, indicating its significant contribution to the prediction results. Followed closely by lag9, which also scored relatively high, suggesting its importance in predicting lung cancer incidence. Other features such as lag3, lag8, and lag0 scored moderately, while the importance scores of lag1, lag6, lag2, lag7, and lag5 gradually decreased, with lag5 scoring the lowest, indicating its minimal impact on the prediction results in the current model.

**Figure 4:**
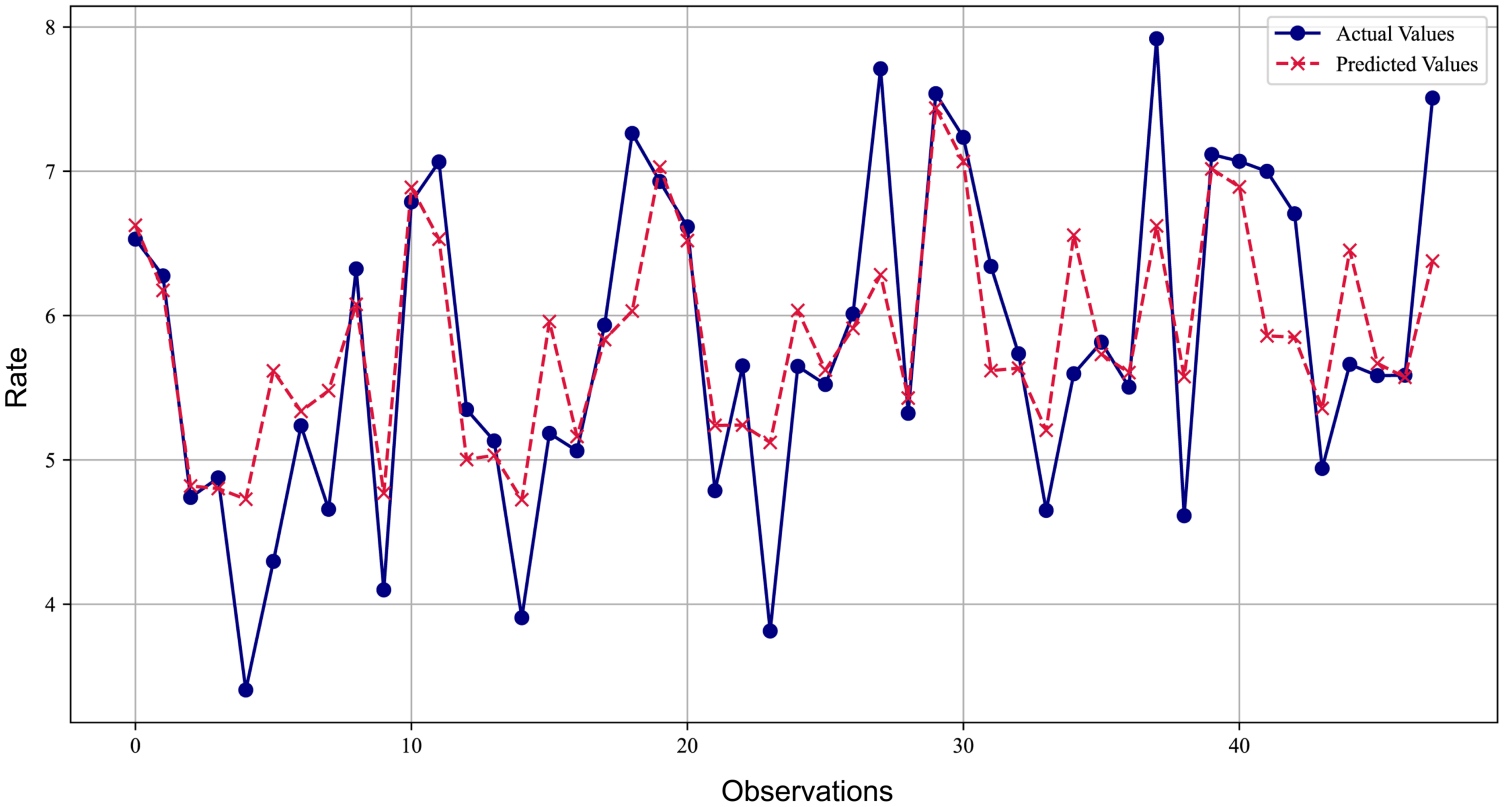
Comparison of SVM model prediction and actual observed values for lung cancer incidence.

Figure 4 compares the predicted values of the SVR model with the actual values. The blue lines and dots represent the actual observations, while the red dashed lines and crosses represent the predicted values of the SVR model. It can be observed from the figure that the model’s predictions are very close to the actual observations at most data points, with the average relative error less than 10%. The error ratios of most data points are concentrated in a lower range (below 5%), reflecting the model’s good predictive accuracy at most data points.

#### 3.2.4 Prediction of lung cancer incidence rate model based on BP-ANN

As shown in Supplementary Table S8, the model performed best with a node count of 7 in the hidden layer, achieving the lowest MSE value.

**Figure 5:**
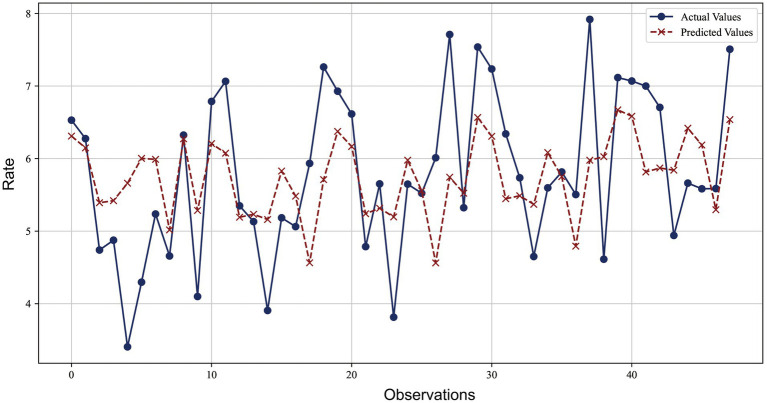
Comparison of BP artificial neural network model prediction and actual observed values for lung cancer incidence.

Figure 5 presents a comparison between the predicted values of the BP-ANN model and the actual values. The blue lines and dots represent the actual observations, while the red dashed lines and crosses represent the predicted values of the model. It can be observed from the figure that the model’s predictions are very close to the actual observations at most data points, with an average relative error of less than 15%. The majority of error values are concentrated in a lower range, further confirming the effectiveness of the BP-ANN model in prediction. However, there are also some larger error values, suggesting that we need to pay attention to and reduce these larger prediction errors in further improvements to the model to enhance its overall predictive performance.

#### 3.2.5 Combined prediction model for lung cancer incidence rate

In this study, we constructed three combined prediction models by assigning different weights to the aforementioned individual models (ridge regression, SVR, and BP-ANN) using the standard deviation method, reciprocal variance method, and optimal weighting method. The detailed weight distribution is presented in Supplementary Table S9.

As shown in the comparison of model performance results in Table 2, the combined model using the optimal weighting method exhibited the best performance across all evaluation metrics. It achieved an MAE of 0.434, MSE of 0.310, MAPE of 7.72%, and a Theil’s U statistic of 0.0475, indicating a high level of predictive accuracy and reliability of the combined model.

**Table 2:** Performance comparison of different prediction models and their combination methods.

Model type	MAE	MSE	MAPE (%)	Theil’s U statistic
Ridge regression	0.7668	0.8290	14.2115	0.0777
Support vector machine	0.4828	0.4343	9.0163	0.0560
BP artificial neural network	0.4449	0.3215	7.8682	0.0484
Standard deviation (combination)	0.5602	0.4953	10.3766	0.0600
Reciprocal of variance (combination)	0.6261	0.5850	11.6066	0.0653
Optimal weighting (combination)	0.4339	0.3104	7.7227	0.0475

### 3.3 Discussions

Previous studies on the association between PM_2.5_ exposure and lung cancer risk usually relied on the average exposure level within a fixed time period, and the selection of exposure years in different studies often differed greatly, which may lead to greater heterogeneity and may mask the true temporal and cumulative effects of PM_2.5_ exposure on lung cancer risk (19–21). This study preliminarily revealed the epidemiological association between long-term air pollution and lung cancer incidence by accurately matching annual PM_2.5_ concentrations in the past 10 years with annual lung cancer incidence data in representative cities in Jiangsu Province. Through lag effect analysis, we found that the strength of the association between PM_2.5_ exposure and lung cancer incidence showed significant time-dependent characteristics. Univariate correlation analysis showed that several lagged years (such as lag3, lag4, lag5, lag7, lag8, and lag9) were significantly associated with lung cancer incidence, with lag9 having the strongest correlation. However, some years (such as lag6) did not reach statistical significance, indicating that the association between PM_2.5_ exposure and lung cancer incidence in a single lagged year was not stable. This finding is consistent with the conclusions of Chen et al.’s spatial epidemiological study using geographically weighted regression, whose results showed significant annual fluctuations in the explanatory power of multi-year PM_2.5_ for lung cancer incidence (22). In addition, univariate analysis may not be sufficient to fully capture the complexity of the effects of PM_2.5_ on lung cancer development. In contrast, gray correlation analysis, which evaluated the overall temporal pattern, showed that there was a consistent strong association in all lagged years, with lag3, lag8, and lag9 showing the highest association levels. These findings suggest that the health effects of PM_2.5_ exposure span many years and reflect long-term cumulative risks rather than individual effects at specific time points, supporting the hypothesis that air pollution has a potential cumulative effect on lung cancer (9, 23, 24). This also highlights the importance of the lag effect in the association between PM_2.5_ and lung cancer incidence, indicating that PM_2.5_ exposure levels at different historical time points are an important predictor of current lung cancer risk. This lag effect may be related to the gradual accumulation of chronic inflammatory response, oxidative stress, and genetic damage in the lungs after PM_2.5_ exposure, which ultimately leads to carcinogenesis many years later (2, 25).

When further exploring the comprehensive impact of PM_2.5_ exposure on lung cancer incidence at different historical time points, this study found significant collinearity problems in the lagged variables through correlation analysis. To address this problem and improve the prediction performance of the model, this paper used robust machine learning methods such as ridge regression, SVR and BP-ANN for modeling. The model results show that machine learning methods can effectively deal with multicollinearity problems and provide relatively accurate prediction results. In the ridge regression model, when the regularization parameter k = 100 was selected, the model showed good stability and fit, and significantly reduced the impact of multicollinearity on the results. The SVR model performed better than other kernel functions after using the RBF kernel function, and could better capture the nonlinear characteristics of the data. In particular, the lag4 and lag9 variables showed high importance in multiple models, further verifying the long-term cumulative effect of PM_2.5_ exposure on lung cancer incidence at different lag periods. The BP-ANN model has great potential in prediction accuracy. Although some data points have large errors, the overall prediction error is small, indicating that it has strong prediction ability.

However, each of these single models has its limitations. Ridge regression’s primary drawback lies in its sensitivity to the ridge parameter, whose prediction performance depends on its selection. Although the optimal k-value can optimize predictions, the choice of this parameter is still controversial and can be influenced by subjective judgments, thereby affecting the prediction results (26). However, each of these single models has its limitations. Ridge regression’s primary drawback lies in its sensitivity to the ridge parameter, whose prediction performance depends on its selection. Although the optimal k-value can optimize predictions, the choice of this parameter is still controversial and can be influenced by subjective judgments, thereby affecting the prediction results (27). While BP-ANN excels at handling nonlinear relationships, its complex structure often leads to overfitting and requires a large amount of data (28, 29).

Combination forecasting models have been proven to effectively improve prediction accuracy in various fields, such as financial market forecasting (30, 31), hydroclimatic forecasting (32, 33), and health risk prediction (9, 34). By integrating the advantages of multiple models, combination models can enhance the robustness and accuracy of predictions (30). This study constructed a combination forecasting model by integrating the results of single prediction models to leverage the strengths of each model while reducing their uncertainties and biases. The integrated prediction model performs best by combining the prediction capabilities of each single model through weighted averaging. In particular, after adopting the optimal weighting method, the integrated model showed higher prediction stability and accuracy, providing a more reliable tool for long-term risk assessment of PM_2.5_ exposure and lung cancer incidence.

Overall, the machine learning model effectively solves the multicollinearity problem in the traditional regression model and can better capture the complex nonlinear relationship between PM_2.5_ exposure and lung cancer incidence. Future research can combine more environmental factors and individual health data to further optimize and verify the predictive ability of these models, and provide a more scientific basis for public health policies and preventive measures.

Although this study provides some insights into the epidemiological relationship between long-term PM_2.5_ exposure and lung cancer incidence, several limitations should be addressed in future studies. First, the geographical scope of this study was limited to representative cities in Jiangsu Province, which may limit the generalizability of the findings to other regions with different environmental and social factors. In addition, the analysis focused on PM_2.5_ exposure and lung cancer incidence, and other potential risk factors such as individual smoking, occupational exposure, and genetic predisposition were not included. Inclusion of specific individualized factors in future studies may improve the accuracy of predictions. In addition, this study considered a 10-year lag period, while longer exposure periods may also significantly affect lung cancer risk, indicating the need for further exploration of extended lag periods. Future studies should also expand their geographical scope to include different regions, integrate more environmental and personal health data, and incorporate real-time monitoring and genomic data. These steps will improve model accuracy, provide a more comprehensive understanding of lung cancer risk, and provide more personalized risk assessments, ultimately contributing to the development of more effective public health strategies.

## Data Availability

The data analyzed in this study is subject to the following licenses/restrictions: none. Requests to access these datasets should be directed to 1053164176@qq.com.
