# Supplementary material for: Epidemiological association and machine learning-based prediction of lung cancer risk linked to long-term lagged satellite-derived PM2.5 in China
Source: Front Public Health. 2025 May 30;13:1536509. doi: 10.3389/fpubh.2025.1536509 (PMC12162561; doi:10.3389/fpubh.2025.1536509)
Supplement: Supplementary file 1 [file Table_1.docx]

Table S1. Shapiro-Wilk Test for Lung Cancer Incidence Rate

| Variable | W | P-value |
| --- | --- | --- |
| rate | 0.979 | 0.549 |
| lag9 | 0.974 | 0.350 |
| lag8 | 0.971 | 0.273 |
| lag7 | 0.972 | 0.294 |
| lag6 | 0.98 | 0.562 |
| lag5 | 0.978 | 0.488 |
| lag4 | 0.975 | 0.393 |
| lag3 | 0.976 | 0.436 |
| lag2 | 0.971 | 0.284 |
| lag1 | 0.965 | 0.159 |
| lag0 | 0.970 | 0.243 |

Table S2 Results of Correlation Analysis for Lung Cancer Incidence

| Variable | Pearson Correlation | | Variable | Pearson Correlation | |
| --- | --- | --- | --- | --- | --- |
|  | *r* | *p* |  | *r* | *p* |
| lag9 | 0.594 | <0.001 | lag4 | 0.560 | <0.001 |
| lag8 | 0.466 | 0.001 | lag3 | 0.488 | <0.001 |
| lag7 | 0.304 | 0.036 | lag2 | 0.265 | 0.069 |
| lag6 | 0.244 | 0.095 | lag1 | 0.014 | 0.927 |
| lag5 | 0.313 | 0.030 | lag0 | -0.018 | 0.905 |

Table S3 Results of Correlation Analysis for Influencing Factors of Lung Cancer Incidence

|  | lag9 | lag8 | lag7 | lag6 | lag5 | lag4 | lag3 | lag2 | lag1 | lag0 |
| --- | --- | --- | --- | --- | --- | --- | --- | --- | --- | --- |
| lag9 | 1 | 0.646** | 0.468** | 0.270 | 0.496** | 0.787** | 0.716** | 0.234 | -0.149 | -0.212 |
| lag8 | 0.646** | 1 | 0.662** | 0.625** | 0.259 | 0.602** | 0.770** | 0.765** | 0.391** | 0.252 |
| lag7 | 0.468** | 0.662** | 1 | 0.744** | 0.634** | 0.332* | 0.571** | 0.716** | 0.691** | 0.587** |
| lag6 | 0.270 | 0.625** | 0.744** | 1 | 0.519** | 0.467** | 0.393** | 0.703** | 0.795** | 0.794** |
| lag5 | 0.496** | 0.259 | 0.634** | 0.519** | 1 | 0.528** | 0.472** | 0.132 | 0.375** | 0.463** |
| lag4 | 0.787** | 0.602** | 0.332* | 0.467** | 0.528** | 1 | 0.602** | 0.232 | -0.016 | 0.041 |
| lag3 | 0.716** | 0.770** | 0.571** | 0.393** | 0.472** | 0.602** | 1 | 0.544** | 0.253 | 0.169 |
| lag2 | 0.234 | 0.765** | 0.716** | 0.703** | 0.132 | 0.232 | 0.544** | 1 | 0.749** | 0.579** |
| lag1 | -0.149 | 0.391** | 0.691** | 0.795** | 0.375** | -0.016 | 0.253 | 0.749** | 1 | 0.951** |
| lag0 | -0.212 | 0.252 | 0.587** | 0.794** | 0.463** | 0.041 | 0.169 | 0.579** | 0.951** | 1 |

** P＜0.01

Table S4 Analysis Results of Tolerance and Variance Inflation Factor (VIF) for Each Influencing Factor

| Variable | Tolerance | VIF |
| --- | --- | --- |
| lag9 | 0.059 | 16.92 |
| lag8 | 0.108 | 9.237 |
| lag7 | 0.100 | 9.952 |
| lag6 | 0.070 | 14.384 |
| lag5 | 0.084 | 11.896 |
| lag4 | 0.100 | 10.013 |
| lag3 | 0.172 | 5.825 |
| lag2 | 0.061 | 16.335 |
| lag1 | 0.014 | 69.888 |
| lag0 | 0.018 | 55.649 |

Table S5 Analysis Results of Eigenvalues and Condition Index for Collinearity Diagnosis

| Dimension | Eigenvalue | Condition Index |
| --- | --- | --- |
| 1 | 10.959 | 1.00 |
| 2 | 0.025 | 20.77 |
| 3 | 0.007 | 39.51 |
| 4 | 0.003 | 64.22 |
| 5 | 0.003 | 65.52 |
| 6 | 0.002 | 82.55 |
| 7 | 0.001 | 137.93 |
| 8 | ＜0.001 | 185.02 |
| 9 | ＜0.001 | 218.26 |
| 10 | ＜0.001 | 273.28 |
| 11 | ＜0.001 | 322.63 |

Table S6 Prediction Coefficients and Modified Variance Inflation Factor (VIF) of Ridge Regression Model (k=100)

| Variable | Coefficient | Modified VIF |
| --- | --- | --- |
| lag0 | -0.029 | 3.43 |
| lag1 | -0.024 | 4.42 |
| lag2 | 0.040 | 2.99 |
| lag3 | 0.084 | 2.33 |
| lag4 | 0.120 | 2.16 |
| lag5 | 0.048 | 1.91 |
| lag6 | 0.021 | 3.59 |
| lag7 | 0.032 | 3.17 |
| lag8 | 0.077 | 3.21 |
| lag9 | 0.126 | 3.01 |

Table S7 Comparison of Performance of Support Vector Machine (SVM) Models with Different Kernel Functions

| Category | MSE | R^2^ |
| --- | --- | --- |
| Linear Kernel | 2.6861 | -0.5603 |
| Sigmoid Kernel | 1.8743 | -0.0887 |
| RBF Kernel | 0.8860 | 0.4854 |
| Polynomial Kernel | 3.6491 | -1.1196 |

Table S8 Comparison of MSE under Different Numbers of Hidden Layer Nodes

| Number of Nodes | MSE | Number of Nodes | MSE |
| --- | --- | --- | --- |
| 5 | 0.6306 | 13 | 0.6476 |
| 6 | 1.7444 | 14 | 0.7455 |
| 7 | 0.5772 | 15 | 1.2197 |
| 8 | 1.5033 | 16 | 0.7855 |
| 9 | 1.1301 | 17 | 0.7104 |
| 10 | 1.7963 | 18 | 0.9674 |
| 11 | 0.9560 | 19 | 1.1857 |
| 12 | 0.6328 | 20 | 0.8985 |

Table S9 Weight Allocation of Different Single Prediction Models under Three Combination Methods

| Model Type | Standard Deviation | Reciprocal of Variance | Optimal Weighting |
| --- | --- | --- | --- |
| Ridge Regression | 0.4858 | 0.6354 | 0.0807 |
| Support Vector Machine | 0.2974 | 0.2381 | 0.3754 |
| BP Artificial Neural Network | 0.2167 | 0.1265 | 0.7240 |
